# Needed independent and dependent variables in multi-element behavior support plans addressing severe behavior problems

**DOI:** 10.1007/s40614-022-00331-4

**Published:** 2022-03-25

**Authors:** Gary W. LaVigna, Elizabeth C. Hughes, Geoff Potter, Matthew Spicer, Linda Hume, Thomas J. Willis, Elena Huerta

**Affiliations:** 1 Institute for Applied Behavior Analysis, Los Angeles, CA USA; 2 The Centre for Positive Behaviour Support, Brisbane, Australia; 3 Possability, Burnie, Australia; 4 Linda Hume Consultancy, Edinburgh, UK

**Keywords:** Multi-element behavior support, Non-aversive reactive strategies, Episodic severity, Strategic capitulation

## Abstract

Ethically , behavior analysts are required to use the least aversive and restrictive procedures capable of managing behaviors of concern. This article introduces and discusses a multi-element paradigm for devising support plans that include ecological, positive programming, and focused-support proactive strategies for reducing the frequency of problem behavior occurrence. It also includes reactive strategies, i.e., separate independent variables. In this paradigm, reactive strategies are aimed solely at getting rapid, safe control over the incident, thereby reducing measured and quantified episodic severity. Behavior analysts who publish in mainstream behavioral journals do not always make it explicit how they, in fact, successfully employed non-aversive reactive procedures to achieve rapid/safe control over the severity of a behavioral incident. Three examples of published studies in the behavioral literature which successfully, though only implicitly, used non-aversive reactive strategies (NARS) to reduce the severity of the behaviors of concern are described. The multi-element paradigm discussed in the present article is illustrated by the support plans that address the challenging behavior of three children in a pre-school setting, using both proactive and reactive strategies. Reactive strategies were used for the purpose of reducing episodic severity (ES) and proactive strategies were aimed at reducing the frequency of occurrence. Following a comprehensive functional analysis and assessment (CFA) and the implementation of a multi-element behavior support (MEBS) plan, results show successful outcomes without the need for any aversive or restrictive procedures. When addressing severe behaviors of concern, in addition to reducing behavioral occurrence, safety should also be improved by reducing ES as a measured outcome and as a function of the reactive strategies employed, including in many cases, the use of strategic capitulation, i.e., providing the identified reinforcer for the target behavior.

## Introduction

Those who support people who exhibit behaviors of concern may sometimes find it necessary to use restrictive procedures to meet one of their major responsibilities, typically referred to as “duty of care.” This important responsibility is to keep people safe, including the person who exhibits the behavior, family members, staff who work with them and the general public. Restrictive procedures include, but may not be limited to, manual or mechanical restraint, physical restraint, seclusion and certain PRN psychotropic medications (i.e., chemical restraint). While it is recognized that such restrictive procedures may be necessary on occasion, there is general concern that they may be over-used and misused. Over-use and misuse have often led to scandal, which has led to state-wide and agency-level practice guidelines and policies aimed at protecting a very vulnerable population. Further, such scandals and the resulting guidelines and policies are not limited to the United States (Weiser, 2020) but are of significant international concern as well, e.g., the United Kingdom (Richards, 2020) and Australia. In Australia, for the 12-month period ending June 30, 2021, the NDIS Quality and Safeguards Commission posted on the web that it had received 1,044,851 reportable incidents; 98.7% of these reports related to the use of an unauthorized restrictive practice (URP) on a person with disability, with 93% of the URPs relating to chemical or environmental restraints (https://www.ndiscommission.gov.au/media-release/3296).

A major application of behavior analysis is to provide support plans that remove the barriers for a person to have a good quality of life caused by behaviors of concern (O’Brien & O’Brien, 1991). Behavioral plans that accomplish that are considered to have produced valued outcomes and to have clinical validity (Favell et al., 1982). Further, the ethics of applied behavior analysis (ABA) requires that this be accomplished using the least-restrictive methods possible. Specifically, the Behavior Analysis Certification Board (BACB, n.d.) states that “The behavior analyst recommends reinforcement rather than punishment whenever possible… Behavior analysts review and appraise the restrictiveness of procedures and always recommend the least restrictive procedures likely to be effective”.

While this ethical principle usually refers to the behavioral procedures of reinforcement and punishment, it can be understood to mean, more broadly, the behavior analyst should recommend non-aversive rather than aversive procedures whenever possible. In fact, specifically with reference to restrictive procedures, the Association for Behavior Analysis International (ABAI) placed on their website in 2010 a statement on restraint and seclusion noting that “ABAI supports the position that treatment selection should be guided by the principle of the least restrictiveness.”

The field recognizes that restrictive procedures are overused and often misused in schools, institutions, group homes, and other treatment settings. This article identifies what we believe to be largely underutilized and unrecognized contributions the science of behavior can make in reducing the use of restraint, seclusion, and other restrictive practices. Firstly, this includes a way of measuring the effectiveness of any reactive strategy, whether aversive or non-aversive, in rapidly and safely bringing a potentially dangerous and harmful behavioral episode under control. Secondly, we will describe the range of largely underutilized, effective, non-aversive reactive strategies (NARS) that can provide rapid, safe situational management.

## Applied behavior analysis (ABA)

### The role of episodic severity in assessing behavioral support

**Fig. 1:**
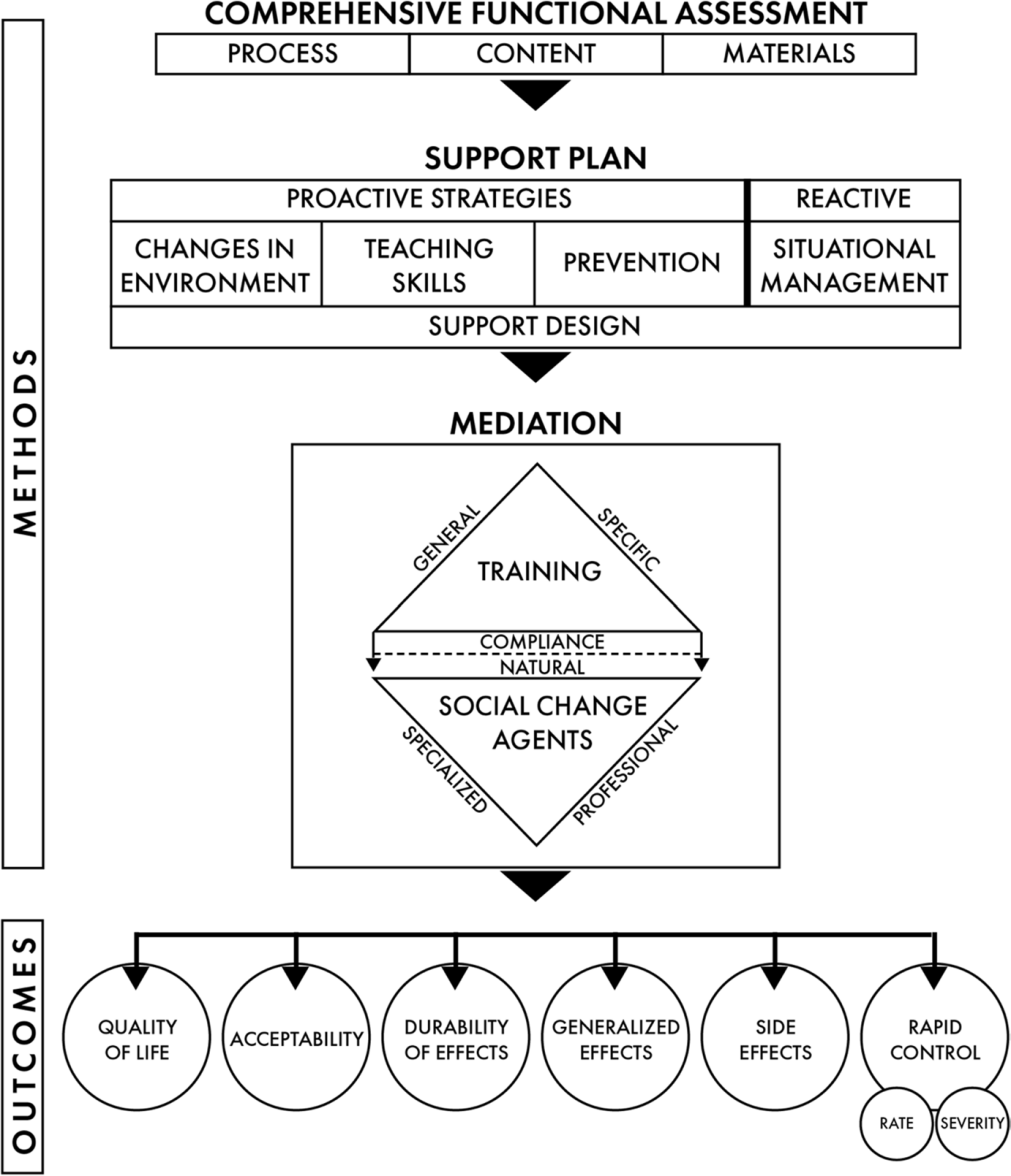
Multi-element behavior support (MEBS) paradigm

ABA is non-linear and multi-element in practice (Layng, 2009). The multi-elements focused on in this article are portrayed in Fig. 1 below, as introduced by LaVigna and Willis (2005b). To begin with, support plans based on ABA have multiple objectives (Baer et al., 1968; Favell et al., 1982; LaVigna & Willis, 2005a; Wolf, 1978). These include, primarily, the outcome of improving the person's quality of life. When this is achieved, the plan is said to have clinical validity. Typically, ABA is used to achieve this through the removal of identified behaviors that are creating a barrier to this outcome. This means rapidly reducing the rate and ultimately eliminating the occurrence of the behavior. However, as important as this is, ABA has recently added an additional outcome measure (ABAI, 2010; LaVigna & Willis, 2005a). This is referred to as episodic severity (ES), defined as the quantified measure of the intensity or gravity of a behavioral incident. The behavior plan should include reactive strategies that resolve the behavioral episode at the lowest level of severity possible.

For example, a quantified measure of the ES of "tantrum" behavior may be the duration of the incident from defined onset to defined offset. Therefore, each week, our behavioral graph can show not only how many tantrums occurred that week but also, for each week, the average level of ES and the range, as measured by duration. Accordingly, our plan can then take responsibility not only for reducing the rate of tantrum behavior but also the average level of ES and the top of the range.

Note the distinction between measuring the ES of behavior vs. measuring severity over time (e.g., Fahmie et al., 2020; Iwata et al., 1990; Sansone & Sansone, 2010). For example, our plan would not be likely to have social validity, i.e., acceptability, if it reduced tantrums from an average of four times a day, for a total of 60 min of tantrums a day, with an average ES level of 15-min duration per episode, to a rate of one time a day, for an average duration total of 30 min per episode. While we would have reduced the rate of tantrums per day from four to one and would have reduced the total duration of tantrums from a total of 60 min a day to 30 min a day, we would have increased the ES of tantrums from an average 15-min duration to twice that length, i.e., to an average of 30 min per tantrum.

Assuming the rate of the target behavior is being reduced, if the ES of the behavior is being reduced, severity over time is also being reduced. However, if severity over time is being reduced, this doesn't necessarily mean that ES is being reduced. Accordingly, when behavioral severity is a concern, support plans should measure ES as an important dependent variable and include reactive strategies aimed at reducing ES.

There is no one way to quantifiably measure ES. It would depend on the behaviors of concern. For example, if property destruction were involved, one measure of ES might be the cost of repair and replacement resulting from the episode, while another could be measuring the duration of the episode from defined onset to offset. That is, there may need to be more than one measure of ES used to measure the effectiveness of the plan's reactive strategies, including the use of restrictive practices if it is deemed to be necessary to include them as part of the plan.

What is needed is an understanding of behavioral procedures defined not only by their future effects but also by their situational effects (LaVigna & Willis, 2005a). Two such procedures (along with others) include the procedures of "resolution," operationally defined as the reactive introduction (positive resolution) or withdrawal (negative resolution) of a stimulus or event that results in an immediate decrease in the likelihood of response continuation or escalation. In contrast, the procedure of "escalation" is operationally defined as the reactive introduction or withdrawal of a stimulus or event which results in an immediate increase in the likelihood of response continuation or escalation (including, therefore, the procedures of both type 1 and type 2 escalation). When a behavior of concern occurs, the behavior plan should include reactive strategies that resolve the episode. It should *not* include strategies that escalate it.

As indicated in Fig. 1, there are also additional desired outcomes for a behavior support plan. They include producing lasting results that generalize across settings, with minimum, if any, negative side effects, and using procedures that are acceptable to the person, responsible family members, support staff and relevant others (i.e., a behavior support plan with social validity). Invariably, this requires plans composed of multiple procedures, as no single procedure can produce this broad and important range of outcomes. These are referred to as multi-element behavior support (MEBS) plans.

## A paradigm for multi-element behavior support (MEBS)

Figure 1 provides a paradigm including all the elements that may be needed for supporting people whose challenging behavior acts as a barrier to their having a good quality of life. To summarize, it is based on the broad range of outcomes sought through the MEBS plan. To achieve those outcomes, the plan is based on a CFA. The resulting MEBS plan includes ecological strategies, i.e., environmental changes that are primarily establishing operations, setting events, and potentiating variables (Leigland, 1984), teaching skills through positive programming strategies (primarily developing alternative sets by increasing the person's behavioral repertoire), focused support strategies (directly preventing or sharply reducing the occurrence of the behaviors of concern) and, when needed, reactive strategies for rapid, safe situational management (reducing ES by using resolution procedures).

MEBS plans are based on a broad functional analysis and assessment (Iwata & Dozier, 2008). In addition to identifying the antecedents and consequences associated with both the high likelihood and low likelihood of the behavior, a functional assessment also identifies the establishing operations, history, skill deficits, health, services received (both presently historically) and other variables that affect the behavior. The contents of a CFA (Willis et al., 2011) are outlined in Table 1.

**Table 1:** Comprehensive functional assessment contents

A. Referral Information
B. Description of The Person 1. Physical Characteristics 2. Cognitive Abilities 3. Communication Abilities 4. Motor/Perceptual Abilities 5. Self-Care Skills 6. Social Skills 7. Community Skills 8. Domestic Skills 9. Leisure/Recreation Skills
C. Other Background Information 1. Family History and Background 2. Living Arrangement 3. Program Placement 4. Health and Medical Issues 5. Service History
D. Mediator Analysis
E. Motivational Analysis
F. Functional Analysis of Behavior 1. Description of Problems 2. History of Problems 3. Antecedent Analysis 4. Consequence Analysis 5. Ecological Analysis 6. Impressions and Analysis of Meaning

### Ecological strategies

Based on the CFA, an MEBS plan is recommended. As indicated in Fig. 1, this includes recommendations for changes in the physical, interpersonal, and service environments (i.e., ecological strategies) aimed at removing the mismatches the CFA has identified between these environments and the person's needs and characteristics. In behavioral terms, these typically involve establishing operations, setting events, and potentiating variables (Leigland, 1984) that have an impact on the target behavior. An example (LaVigna & Willis, 1992) of needing a CFA going beyond a functional analysis in order to develop an effective MEBS plan that included ecological strategies involved serious self-injurious behavior. In addition to being on the autism spectrum, the teenage boy was also deaf due to maternal rubella. A tremendous mismatch existed in the service environment in that while he was learning to communicate using sign language, his support staff was not fluent in sign language, nor were the peers he was living with. One of the ecological recommendations made in his support plan was that he live with peers and be supported by staff who were fluent in sign language.

### Positive programming strategies

MEBS plans based on a CFA also include recommendations for teaching certain identified functional, chronologically age-appropriate skills using specifically recommended teaching procedures to increase the person's behavioral repertoire and alternative sets (Goldiamond, 1975), i.e., positive programming (LaVigna et al., 1989). Positive programming involves teaching four categories of skills, general, functionally equivalent, functionally related, and coping/tolerance skills. General skills give the person more independence involving day-to-day activities such as cooking, cleaning, bathing, shopping and other selfcare, home care, and community skills. Skills in this category also include those the person would very much like to learn, e.g., how to independently play music of their choice on a streaming device or how to independently access a website of choice.

Positive programming also includes teaching the person functionally equivalent skills, that is, socially acceptable behaviors that successfully serve the same function as the behavior of concern. For example, this might involve teaching the person to use their communication system (e.g., the spoken word, sign language, communication cards, or PEC symbols) to communicate important things such as "no, I don't want to," "I'm confused, I don't understand what you want me to do," "can you help me," etc.

In addition, positive programming includes teaching skills that are functionally related to the behavior of concern. This may include, for example, discrimination skills such as being able to discriminate edible from inedible items or socially acceptable interactions from socially unacceptable interactions. An exceptionally important functionally related skill, if the person has not already mastered it, is to be brought under generalized instructional control when reasonably asked to do something. That is, if the person is asked to do something reasonable and in context, that they understand and know how to do, they are highly likely to do it.

Finally, the last category of positive programming is to teach the person to tolerate and cope with naturally occurring aversive events that are a part of everyday life. This focuses on such events that are frequent antecedents to the behaviors of concern. Examples of such events include having to wait for something (e.g., waiting for dinner to be ready), having to do something you don't want to do (e.g., doing your fair share of household chores), or receiving any criticism from another person. One way to teach someone how to tolerate a naturally occurring aversive event is to "start small & move slow." For example, in teaching someone how to tolerate waiting for something they have asked for, we might first ask them to "please wait" (with a brief explanation as to why), e.g., "please wait while I get it," or "please wait until its ready." Initially, the wait time might only be a few seconds but with three successes in a row without the behaviors of concern, we would gradually increase the waiting time until the person is able to reasonably wait for anything, regardless of how long. In fact, in one of our cases where waiting was a primary issue, he got to the point where he said on one occasion that he wanted to go swimming, and staff said, “it’s February and it’s too cold to go swimming, you'll have to wait until June,” which he was able to do without exhibiting challenging behavior.

### Focused support strategies

Ecological strategies (e.g., being with people who understand your language of communication, having a say as to who you are going to live with and having a defined minimum frequency of time-based preferred events in your life) are likely to be permanently needed and may themselves represent direct improvements in the person's quality of life. That is, in the MEBS paradigm, improved quality of life is both the primary outcome objective and a support strategy. Further, functional skills learned through positive programming are likely to be permanent additions to the person's behavioral skill repertoire, given the natural reinforcement intrinsic to those "alternative sets" of behavior, i.e., alternatives to the behaviors of concern. While some of these ecological and positive programming strategies may have immediate effects on the behaviors of concern, it is also important for an MEBS plan to include focused support strategies (LaVigna & Donnellan, 1986; LaVigna & Willis, 2005b) for the specific purpose of rapidly reducing and ultimately preventing the occurrence of the behaviors of concern.

Unlike the ecological strategies, focused support strategies are only needed temporarily while the long-term goals of the ecological and positive programming strategies are being pursued. They include, for example, antecedent control strategies, the purpose of which is to eliminate or minimize the presence of those discriminative stimuli associated with the higher likelihood of target behavior occurrence. This would be until the person has been taught to cope with, and tolerate their presence, without exhibiting those behaviors of concern or until these antecedents can be eliminated from the person's life totally and permanently. Antecedent control can also include increasing the presence of those discriminative stimuli associated with behaviors that are alternatives to the behaviors of concern, i.e., associated with the lower likelihood of challenging behavior.

Focused support strategies in an MEBS plan might also include stimulus satiation through non-contingent access to the reinforcer motivating the behavior. This abolishing operation would be aimed at producing a level of access to the preferred event that reduces the person's motivation to seek more of it through exhibiting the target behavior, thereby reducing the occurrence of that behavior. Such stimulus satiation may need to be maintained until the person is taught a functionally equivalent skill through positive programming, i.e., being taught a more socially acceptable way of acquiring the preferred event without exhibiting the behavior of concern, e.g., simply asking for it.

Schedules of reinforcement represent yet a further example of focused support strategies aimed at reducing and, if possible, eliminating the occurrence of the target behavior (LaVigna & Donnellan, 1986; LaVigna & Willis, 2005c). These include differentially reinforcing and increasing alternative behaviors (DRA), differentially reinforcing incompatible behaviors (DRI), differentially reinforcing low rates of the behaviors of concern (DRL), differentially reinforcing other behavior (DRO), and differentially reinforcing other behavior with progressively increased reinforcement (DROP), especially effective with low-frequency behaviors.

### Reactive strategies

Ecological, positive programming and focused support strategies are all aimed toward the future, i.e., giving the person a good quality of life without the barriers represented by the occurrence of the challenging behavior. Accordingly, these are referred to as proactive strategies. However, should the behaviors of concern occur, and if those behaviors are putting the person or others at risk, the primary objective is rapid, safe situational management to keep everyone free from harm. Accordingly, the role of a reactive strategy in the MEBS plan is to reduce the ES of the behavior, an important but typically unmeasured dependent variable, i.e., outcome measure.

Reactive strategies for the purpose of rapid, safe situational management resulting in the measured reduction of ES of the behavior through its resolution include, for example, redirection to an alternative response. A good example of this is to redirect the person to engage in the functionally equivalent skill that he or she is being taught through positive programming. Another example of a reactive strategy is the use of stimulus change or props (Azrin, 1958; Niepel, 2001). This is defined as the non-contingent and sudden introduction of a novel stimulus or a dramatic alteration of the incidental stimulus conditions, which results in an immediate reduction in ES. Examples may include support staff breaking into song and dance or using a remote control to click on a prepared device to stream certain music throughout the house, e.g., the William Tell Overture. This takes advantage of the "stimulus props" and can help avoid the need for restrictive/aversive reactive strategies that may themselves increase ES.

The use of stimulus change is not typically the only reactive strategy used but may be a "door opening" strategy. That is, it may be the first step starting with the resolution of the behavioral incident and ending with a return to reengagement with the schedule for the day. Yet another example of a "door opening" reactive strategy might be "active listening" (Gordon, 1970; Royce, 2005). Active listening is defined as reflecting back to the person the message they are communicating through their behavior. This can be done by paraphrasing the message that the person is understood to be communicating, for example: "you seem really upset because it is time for Jane to use the computer" or "you really don't want to stop using the computer." Thomas Gordon (1970) provides guidelines for when it is OK to transition from active listening to redirection, problem solving or some other strategy or activity.

## MEBS implementation

The MEBS paradigm portrayed in Fig. 1 also includes the application of behavior science to assure the consistent implementation, i.e., mediation, of the agreed upon plan (LaVigna et al., 1994). This application, referred to as Periodic Service Review (PSR), includes the clear step-by-step description of how each procedure is to be implemented, the competency-based criterion referenced training of the relevant service providers responsible for the procedure’s implementation, monitoring of the service providers' implementation through formal fidelity checks, and visual feedback graphs over time that show what percentage of the plan is being implemented consistently. Research has shown that such visual feedback graphs motivate staff to improve their performance (Lown et al., 2021; Quilitch, 1975). An unmet criterion is characterized as an opportunity to improve service quality (a good thing) rather than as an indicator of poor performance (a bad thing). (Go to the following link for access to the Excel program that can be used to produce such feedback graphs: < iaba.com/PSRimplementation.xls > .)

## Minimizing the need for restrictive practices

When reactive strategies, including restrictive procedures, are needed to keep people safe, this should not be viewed as needing something separate from the behavior support plan because the plan didn’t work. Rather, in such situations, reactive strategies should be included as part of the plan to keep ES at a minimum. That is, the role of the reactive strategies in an MEBS plan should be to resolve the behavioral incident at the lowest possible level of ES (LaVigna & Willis, 2002, 2005a). Further, given the ethical principle of using the least restrictive and aversive methods capable of solving a behavioral problem, non-aversive reactive strategies (NARS) (Crates & Spicer, 2016; Spicer & Crates, 2016) should be planned and used as a first resort. Aversive, restrictive reactive strategies should only be used as a last resort. The belief that the more severe the behavioral event, the more restrictive you need to be to bring it under control is a fallacy (LaVigna & Willis, 2016). In fact, NARS are more effective than the aversive reactive strategies in minimizing ES (Spicer & Crates, 2016).

Numerous studies have shown that behavioral plans based on functional analyses and assessment are more effective than behavior plans that are non-function based (Hanley et al., 2003). This also extends to reactive strategies. Research shows that function-based NARS are more effective than non-function-based NARS (Spicer & Crates, 2016). In fact, one of the strongest ways of identifying the possible function of a behavior is to determine what causes the behavior to stop. For example, if attending to the person when the behavior occurs always results in a cessation of the behavior, it is likely that such contingent attention is at least one of the reinforcers for the behavior. However, it also indicates that the provision of attention contingent on the behavior of concern has the situational effect of resolution. In devising a behavior support plan for that behavior, in addition to the proactive ecological, positive programming, and focused support strategies, we might very well include giving the person the attention she or he wants, not as a last resort but as a first resort reactive strategy, providing that attention as early in the event as possible in order to minimize ES. This requires, of course, that the other components of the plan, in addition to their other contributions to the long-term outcomes, also provide the positive programming, setting events, establishing operations and potentiating variables (Crates & Spicer, 2016; Leigland, 1984) that would prevent the attention from reinforcing the target behavior. That is, giving the person what they want is not simply capitulation but strategic capitulation (LaVigna & Willis, 2002). Some examples of setting events, establishing operations, and potentiating variables that would prevent the unwanted reinforcement of the behavior of concern could include increasing the density of both time-based and contingency-based preferred events in the person’s life, increasing access to the motivating reinforcer when the behavior does not and is not occurring, prompt identification and effective treatment for medical condition, being able to choose housemates, etc.

Some behavior analysts, not to mention the general public, may be reluctant to react to behavior by giving the person what they want, since they believe that this will just further reinforce it. However, there are multiple examples of behavioral studies reported in ABA-based journals that evaluate the effectiveness of various non-aversive support strategies in reducing the occurrence of the identified target behaviors and, without explicit description, which use NARS to minimize ES, also without explicit measurement. Following are three examples of such studies.

The first study we are referencing was carried out by Slocum and Vollmer (2015). They were investigating “escape behavior,” i.e., not doing what they were requested to do, as exhibited by the five subjects in the study. One of the proactive, focused support strategies being evaluated by the study was using a 30-s break from instruction as negative reinforcement for compliance. The effectiveness of this in increasing compliance was compared with positively reinforcing compliance with an edible treat. Response extinction was not used in either case. That is, if the person was noncompliant to the request being made, a 30-s break was provided. The reason for not using response extinction was to avoid the known side-effect of that strategy, including the possibility of physical aggression, referred to as an “extinction burst.”

The results of this study showed that positive reinforcement was more effective in reducing non-compliance than negative reinforcement for all five of the participants in the study. This gives practitioners in the field useful information. Reading between the lines, however, there are additional findings that represent important contributions. While not explicitly labeled, the NARS strategy of strategic capitulation (reacting to non-compliance by providing a 30-s break) was used to minimize ES. Their stated purpose for reacting to non-compliance with a 30-s break, was to prevent an extinction burst. While not formally measured, it was clear by the authors’ statements that the behaviors associated with an "extinction burst” were avoided, as they had planned. We suggest that the labeling of strategic capitulation as the function-based NARS of negative resolution and graphing its measured effects on ES, without reinforcing the problem behavior, would significantly add to the contribution of this study to the practice of ABA.

The second study we are referencing was carried out by de Zubicaray and Clair (1998). They were evaluating differential reinforcement of other behavior (DRO) and differential reinforcement of incompatible behavior (DRI) as proactive strategies and restitution/reassurance training (RRT) as the reactive strategy when verbally abusive and/or physically aggressive behavior occurred. This involved Carol (the name given to the person of concern) apologizing for disturbing the ward milieu and reassuring all co-patients and staff present that she would not verbally abuse them again, as well as to help the nurse complete the necessary report of the incident. The entire multi-element plan was developed with Carol’s participation and with her informed consent. A functional analysis concluded that attention from staff was the operative positive reinforcer for verbal abuse and aggression. The procedures were integrated into a multi-element support plan for Carol who was diagnosed as having moderate mental retardation. The intervention plan required that if Carol exhibited either verbal abuse or physical aggression, staff immediately attended to her and implemented their protocol for social reassurance training. After the baseline phase of the study, the second phase of the study included DRO, DRI, and RRT. The third phase of the study included DRI and RRT and the fourth phase DRI only.

Since it was staff who immediately implemented the RRT protocol, this amounted to the use of the function-based NARS of strategic capitulation, in this case, positive resolution. Since the function of verbal abuse and aggression was to acquire staff attention, this behavior stopped immediately when staff implemented the agreed upon protocol. Given that verbal abuse always had occurred before physical aggression, with implementation of the RRT protocol, physical aggression no longer occurred. Again, we suggest that the labeling of strategic capitulation as the NARS of positive resolution as a specific component of the plan and graphing its measured effects on ES, without reinforcing the problem behavior, would significantly add to the contribution of this study to the practice of ABA.

In a third behavioral study (Dowdy & Tincani, 2020), two teenage males were treated for their transitional refusal behavior when they were asked and prompted to get out of the swimming pool, go to the locker room, and to get dressed. During baseline, their refusal behavior consisted of any attempt to push away, hit with an open or closed fist, and/or push pull or grab the therapist. In proactive treatment, both teens were instructed and prompted as described above, upon which they were told they would be able to enjoy their favorite music and/or special treats. For compliance, the therapist provided their promised positive reinforcement, i.e., the music and/or special treats. Reactively, if the participant exhibited transitional refusal, immediately “the therapist removed the demand to leave the pool and allowed uninterrupted access to the pool.” That is, the therapist employed negative resolution using the procedure of strategic capitulation. The traditional strategy of extinction was not used. The results were quite impressive. Refusal behavior was rapidly reduced and then eliminated. In addition, the boys were also going to their lockers and getting dressed as requested. Although not directly measured, the data presented clearly indicate that ES was sharply reduced by replacing a traditional extinction procedure with the function-based NARS, strategic capitulation.

Journals not limited to behavior analysis have published studies that address these important behavioral principles and procedures. That is, many of these studies have researched the measured outcomes of resolution using NARS for the purpose of reducing ES without the need for restrictive procedures. For example, in a study of 24 cases reported by Crates and Spicer (2016), the 24 MEBS plans demonstrated significant reductions in occurrence, ES, restraint, and the elimination of seclusion. These outcomes demonstrate the efficacy of NARS for maintaining safety without resorting to aversive or restrictive practices.

In another study (Spicer & Crates, 2016), NARS for reducing the ES of aggression in 17 cases were evaluated. The findings were as follows: when function-based NARS were used, the impact was immediate resolution 100% of the time, that is, aggression stopped. When a non-function-based NARS was used, immediate resolution occurred almost 48% of the time, immediate de-escalation to a lower level of ES occurred 20% of the time, continuation at the same level of ES occurred 25% of the time and escalation to a higher level of ES occurred 7% of the time. In contrast, when an aversive consequence was used, it produced resolution only 10% of the time, never resulted in de-escalation, maintained the same level of ES 42% of the time and escalated the episode to a higher level of ES 48% of the time. When a restrictive procedure was used, neither did it produce resolution, de-escalation nor escalation, but led to the continuation of the same level of ES.

A third publication (Hughes & Huerta, 2016) involved MEBS support plans in four case studies that met Kazdin’s (1981) criteria allowing valid inferences. The interventions took place in classroom settings and involved severe behavior problems such as physical aggression, self-injury, and property destruction. Each of the student’s support plans was based on a CFA and included ecological strategies, positive programming, focused support, and NARS. No aversive or restrictive strategies were used. Data collection for behavioral occurrences were checked and confirmed for reliability and support plan procedures were regularly checked and confirmed for fidelity. The results showed a dramatic reduction in both average level and highest level of ES for all four students. By the end of the school year, the target behavior was no longer occurring for two of the students and was occurring less than once-a-week but at a very low level of ES for the other two.

In summary, while it is clear behavior analysts consider the severity of behavior to be a serious concern that falls under its umbrella (e.g., Iwata et al., 1990), the primary behavior analysis journals have not published studies that have included ES as a quantifiably measured dependent variable. Nor have they published studies that evaluate the effectiveness the independent variables of NARS in reducing episodic severity.

## An example of MEBS including the ES as a dependent variable and NARS as independent variables method

This application of the MEBS paradigm addressed the highly challenging behavior presented by three 4-year-old boys in a pre-school setting. School attendance for each of the three included Monday–Friday for 30.5 h total per week, consisting of four 6.5-h days and one 4.5-h day. Using fictitious names, Cal is of Latinx origin and was diagnosed with autistic spectrum disorder; Omar is also of Latinx origin was diagnosed with oppositional defiant disorder; and Martin is of mixed ethnicity, Caucasian–Latinx, had no formal diagnosis, and was born with drug exposure. From the age of 6 months, he was placed with his grandparents due to parental neglect. All three children had experienced high levels of challenging behavior, including aggression resulting in the need for medical attention in a range of settings, including their homes and previous school placements.

## Service context

All children received services within a classroom-based preschool intensive therapeutic program. Each class had eight children, with between two and four adults depending on the time of day. Within the first week of service initiation, the assigned behavior analyst undertook records reviews, interviews, brief play/interactions with the student and observations. The information gathered was used to define the behavior of concern and establish the function of behavior, develop an initial MEBS plan, and begin collecting data.

## Intervention overview/list of proactive and reactive strategies

Each MEBS plan included both proactive and reactive strategies. Reactive strategies were included for the sole purpose of minimizing ES and achieving rapid and safe resolution of the target behavior when it occurred. We used both function-based and non-function-based NARS, including strategic capitulation, active listening, expressing affection, providing access to a highly preferred person, offering alternative preferred items or special activities, and stimulus change. Proactive strategies were included to reduce occurrence of the target behavior and to prevent any potential counter-therapeutic side effects of the reactive strategies, such as reinforcement of the target behavior. Proactive strategies included ecological strategies for removing any mismatches between the person’s needs and the environment; focused support strategies, including antecedent control techniques and differential schedules of reinforcement, to prevent the occurrence of the target behavior; and positive programming to teach alternative prosocial skills for meeting their needs, communicating and coping. Punishment and other aversive reactive strategies were not used with any of the students. In accordance with the MEBS paradigm, staff were provided with competency-based training for their responsible procedures. Formal and regularly scheduled procedural reliability, i.e., fidelity, checks were performed. These were carried out on a regularly scheduled basis by members of the management team with BCBA, BA MA, or licensed mental health credentials throughout the entire school year. Procedural fidelity scores were consistently above an average of 85%. An outline of the methods included in the MEBS plan is provided in Table 2.

**Table 2:** MEBS plan content

MEBS PLAN	PHASE 1			PHASE 2		
	Cal	Omar	Martin	Cal	Omar	Martin
Ecological Strategies						
Comprehensive Functional Assessment	X	X	X			
Interactional Style Protocol	X	X	X	X	X	X
Free Access to Certain Preferred Items, Etc						
Positive Programming						
General Skills Training:						
Fun: Games & Other Skills the Student Wants to Learn	X	X	X	X	X	X
Useful: Toileting/turn taking/lining up, etc	X	X	X	X	X	X
Functionally Equivalent Skill Training:						
Acceptable ways of getting needs met, e.g., requesting interaction/attention				X	X	X
Functionally Related Skills Training:				X	X	X
For example, accepting alternatives offered when primary desire can’t be met				X	X	X
Coping and Tolerance Skills Training:						
For example, how to ask for help				X	X	X
Focused Support						
Individualized DRO Schedule	X	X	X	X	X	X
Individualized DRA schedule for functionally equivalent and related skills				X	X	X
Stimulus satiation (free access to functional S^R^)				X	X	X
Antecedent control (reduce known triggers and increase those associated with low-rate behavior.)				X	X	X
(In addition, the reactive strategies below were also used as focused support strategies in response to precursor behaviors.)	X	X	X	X	X	X
Reactive Strategies						
Function Based NARS:						
Strategic capitulation, active listening	X	X	X	X	X	X
Non-function Based NARS:						
Stimulus change, redirect to competing preferred items or activities, inter-positioning by using a pillow for protection, etc	X	X	X	X	X	X

“X” indicates full implementation of the procedure. As indicated, the CFA for each student was finished within their first month of enrollment. Accordingly, those procedures requiring the results of the CFA were not fully implemented until the 2nd phase. Those parts of the MEBS plan fully implemented in phase 1 were based on information provided in the referral packet and process

To further describe intervention, equivalent and related skills were practiced between 10 and 30 times per day in role-play or contrived situation opportunities. The students were prompted to use the functionally equivalent skills they were being taught upon demonstration of precursor behavior as a preventative strategy. All three students participated in all of the regular activities of the program day. No activity removal was utilized, though they may have chosen to participate in certain activities over others, as is the norm in a general education preschool context. As opposed to removing any preferred activities, activities that were identified as maintaining events (e.g., access to preferred tangibles, attention, or breaks) were provided at an increased level compared to pre-treatment, on time-based contingencies in order to act as an abolishing operation for those activities acting to reinforce the challenging behavior. Access was determined by establishing levels of free-access for each reinforcing event and setting time-based access at 120% of free access levels (rounded to the next whole number). For example, if a student was given the option to leave lessons at-will and they were noted to leave lessons five times an hour, they were given six opportunities to leave lessons per hour. If a child sought preferred interactions ten times per hour, they were provided with 12 noncontingent preferred interactions per hour. This was intended to serve two purposes: 1) to satiate the student of the reinforcer maintaining the challenging behavior, thereby decreasing its value as a reinforcer and its strength in maintaining challenging behavior. The second purpose was to enable staff to use redirection to preferred events as a de-escalation tool without reinforcing the challenging behavior (as the reinforcing value of the event has been undermined by the noncontingent access to this reinforcer per #1). Contingent schedules of reinforcers (e.g., those used to increase the rate of functional skills) were additive—that is, they were additional items and/or activities provided in the environment, or an enhancement of already existing items/activities. For example, for a student whose challenging behavior was analyzed to be for the function of gaining attention from preferred individuals, that student would be provided time-based access to interactions with those individuals. This was rather than limiting access to interactions with preferred individuals as a contingency for the demonstration of appropriate behavior. Then, to create motivation to demonstrate replacement behaviors and reduce levels of challenging behavior, an enhanced version of those interactions was offered as a contingent reinforcer. Enhancements for an interaction-based reinforcer could include longer duration of the interaction event, “special” activities during interactions, or interactions with multiple preferred people at once.

## Measured outcomes

Based on information obtained during the referral process, data collection began on the student’s first day of school attendance. In each case, the occurrence of behavior was measured by rate, i.e., frequency, per day. The ES of each occurrence was measured by the highest rating of the behavioral incident, based on a ten-point scale. Each student had an individualized scale of ES (see Table 3 below). However, for each, the highest level of ES for an outburst involved physical aggression that resulted in someone needing medical attention. While the targeted behavior for each of the three was “outburst behavior,” the included topographies also varied, as shown in Table 4 below. The onset for recording the occurrence of each student’s behavior was the first occurrence of any topography listed in their defined behavior and offset of the episode was after a specified number of minutes transpired with no target behavior topographies nor precursors. The identified precursors to the target behavior are also shown in Table 4. Finally, the conclusions of each student’s CFA completed in the second month of the student’s attendance regarding the function of their outburst behavior are also shown in Table 4.

**Table 3:** Episodic severity scales for outburst behavior

**Cal** LEVEL1: Attempt at any topography LEVEL2: Contact with a surface, no injury or damage LEVEL3: Property destruction, less than $50 LEVEL4: Property destruction, less than $100 LEVEL5: Aggressive contact, no mark left LEVEL6: Aggressive contact, mark left, no medical attention/first aide needed LEVEL7: Property destruction, over $100 LEVEL8: Aggressive contact, multiple individuals LEVEL9: Aggressive contact, first aid needed LEVEL10: Any topography resulting in need for medical attention
**Omar** LEVEL1: Attempt at any topography LEVEL2: Screaming lasting for less than 5 min LEVEL3: Screaming lasting for more than 5 min LEVEL4: Hiding in a room with a caregiver, being found within 5 min LEVEL5: Aggression, no mark left LEVEL6: Hiding outside of room with caregiver, or not being found within 5 min LEVEL7: Aggression mark left LEVEL8: Aggression towards multiple people, mark left LEVEL9: Aggression requiring first aid LEVEL10: Aggression requiring medical attention
**Martin** LEVEL1: Attempt at any topography LEVEL2: Leaving room, staying in house/building LEVEL3: Leaving house/building LEVEL4: Aggression, no mark left LEVEL5: Aggression, mark left LEVEL6: Leaving house/building and entering area with traffic OR being lost for more than 5 min, less than 15 min LEVEL7: Aggression with multiple people contacted with marks left LEVEL8: Being lost for more than 15 min LEVEL9: Aggression, first aid needed LEVEL10: Aggression, medical attention needed

**Table 4:** Topographies of and precursors to outburst behavior and identified function

**Cal** Topographies Physical Aggression: *hitting, kicking, pinching, pushing* Property Destruction: *breaking any item that renders it useless, throwing items not meant to be thrown, banging on surfaces with body or objects hard enough to make a sound heard across the room* Precursors *Body tensing, growling, grunting, huff/puff breathing, reaching for items, grimace* Function *Wanting something tangible*
**Omar** Topographies Physical Aggression: *hitting, kicking, throwing items at others* Screaming: *saying words or making sounds at high enough volume to be heard in another room through a closed door* Hiding: *going under furniture and curling up into a ball or seeking a confined space to be contained inside of, without others knowing where he is* Precursors *Complaining things are “not fair,” pounding fist on surfaces, bumping into others, kicking legs at the air, making shooting noises with mouth* Functions *Wanting attention or escape*
**Martin** Topographies Physical Aggression: *biting, punching, hitting with objects, kicking, jumping on top of other people to the extent that they fall, grabbing others’ items or clothing* Eloping: *leaving the room where caregiver is located and not returning within 5 s when asked to, “Come back*.” Precursors *Verbal threats of aggression, voice volume increasing, stomping feet, knocking into furniture or people, saying “I hate you,” “you’re stupid,” “you’re a* < *name calling word,* > *profanity, threatening to leave the room, telling someone they are wrong or “picking a fight’ over something seemingly insignificant (e.g., she has curly hair-that is not fair.”* Functions *Wanting something tangible or attention*

Reliability indices for occurrence and ES data for each of the three students were established using monthly inter-rater reliability checks for each student in the class receiving behavioral services, with the class average score required to be over 85%, otherwise triggering an individual plan review. Those responsible for were the school’s paraprofessional staff as well as professional level staff subbing for paraprofessionals. Monthly reliability indices remained above 85% for both occurrence and ES data, with one exception. One student’s initial reliability score on the first day was only 60%.

## Results

**Fig. 2:**
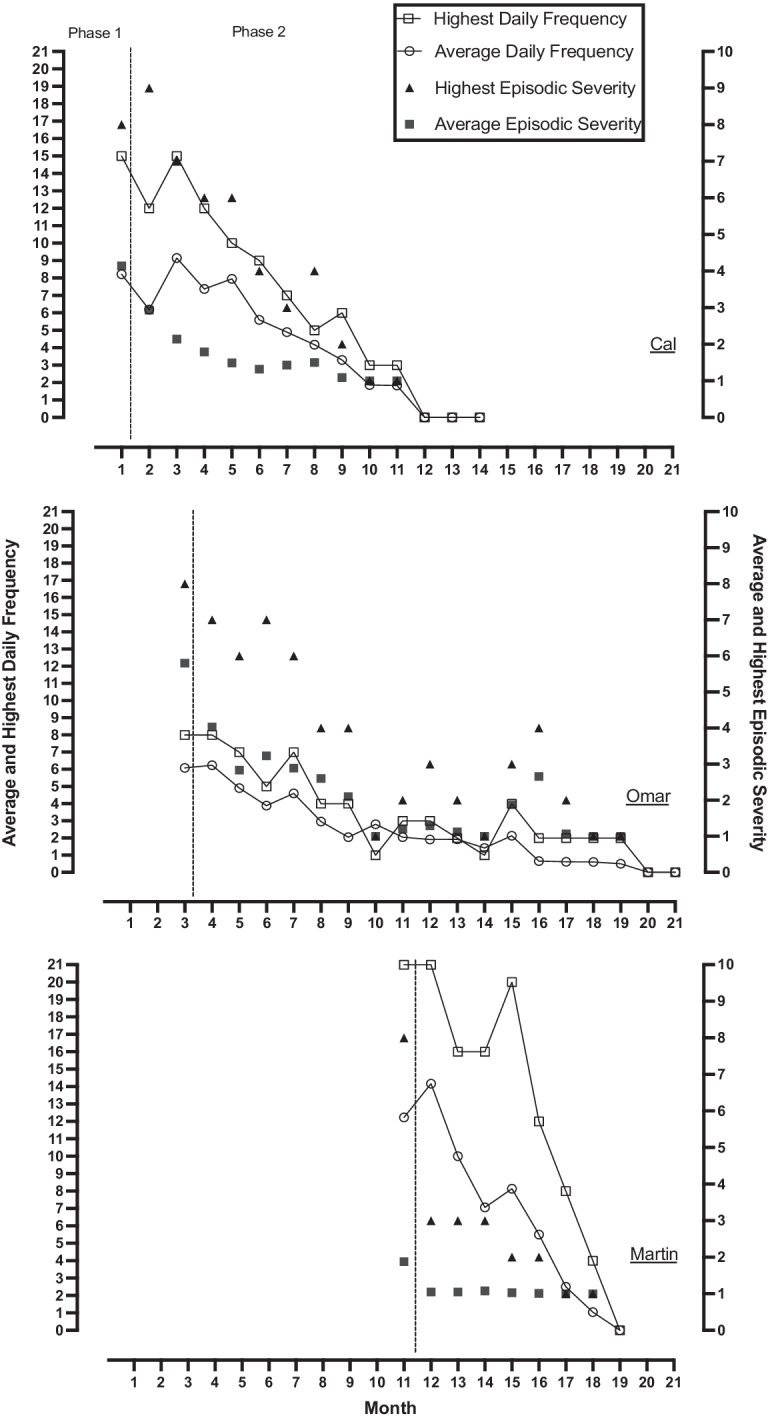
Results of MEBS Plans on Episodic Severity and Frequency of Outburst Behavior

Figure 2 shows the results for Cal’s, Omar’s, and Martin’s MEBS plans for outburst behavior and its ES. It illustrates the highest point of ES and the average ES for the month. It also shows the average and highest daily frequency of outburst behavior for the month for each of the three participants. A score of 0 occurrences for the month indicates no ES measure was recorded since no incidents occurred. Outburst behavior during phase 1 for Cal occurred an average of 8.22 times a day and, in phase 2, reduced to 0 times a day with a corresponding decrease in ES. Level 10 ES outburst behavior never occurred. During phase 1, Omar’s outburst behavior occurred an average of 6.08 times a day and, during phase 2, reduced to zero with a corresponding decrease in ES. Levels 9 & 10 ES outburst behavior never occurred. Outburst behavior during phase 1 for Martin occurred an average of 12.23 times a day and, during phase 2, decreased to zero with a corresponding decrease in ES. Levels 9 & 10 ES outburst behavior never occurred. A highly important result is the highest point of ES for the month. The highest point of monthly ES in Fig. 2 shows a steady decline to a score of 1 across phase 2, if an outburst even occurred, for all participants. A measure of the effectiveness of the reactive strategies for the participants in minimizing the ES is the monthly highest point.

## Conclusions

In the brief example of MEBS provided above, plans effectively reduced the occurrence and ES of outburst behavior without the need for any punishment (which if used in a MEBS plan would be considered both a focused support strategy and a reactive strategy), restrictive or other aversive procedures. The plans explicitly included the reactive strategies of function-based and non-function based NARS specifically aimed at reducing ES. While the multitude of strategies implemented in each of the three case studies precludes the ability to identify the specific causal effect of individual strategies. the plans in their entirety resulted in significant reductions in both the frequency and the ES. Within the context of a MEBS plan, one may be able to react to problem behavior with a non-aversive, even positive event, including giving the person what he or she wants, i.e., strategic capitulation, while also reducing the frequency of the behavior. That is, the delivery of a preferred event in response to problem behavior can have a limited negative impact in the context of a full MEBS plan. The novelty of the MEBS paradigm described used here is that we were unable to find any other application or research study in any issue of the leading ABA journals in which the ES of each occurrence of the target behavior was quantitatively measure as a dependent variable and/or in which one or more NARS strategies were evaluated as independent variables.

We believe the explicit use of NARS (independent variables in behavioral science) reduces quantitatively measured ES (dependent variables in behavioral science) and therefore represents a significant contribution to the practice of ABA with reference to the ethical and legal mandate to eliminate the unnecessary and excessive use of restrictive practices in schools, institutions, group homes, and other service settings. We also believe that this contribution can be greatly increased through the major behavioral journals. These include the *Journal of Applied Behavior Analysis*; *Behavioral Interventions*; *Behavior Analysis in Practice*; *Perspectives on Behavioral Science*; and *Education and Treatment of Children*. Our recommendations include the following:

1. Studies that investigate support plans for challenging behavior that include NARS and outcome measures for ES should be submitted to one of the major applied behavior analysis journals.
2. Such studies should investigate not only the effects of full applications of the MEBS paradigm on the full range of the desired ABA outcomes but also the specific contribution made by specific elements of the plan to those outcomes.
3. The mentioned journals should require that studies investigating support plans for challenging behavior should also include the use of NARS as a first resort and that those studies include outcome measures for ES as well as for occurrence.
4. Studies would be useful that sample or survey the measurement of ES and use of NARS by behavior analysts practicing in the field.
5. Behavioral science studies that compare the relative effectiveness of different NARS in reducing ES, which comparisons were not possible in the three case studies reported here, would be very useful to ABA practitioners.
